# The modulation of grain boundary barrier in ZnMgO/ZnO heterostructure by surface polar liquid

**DOI:** 10.1038/srep04185

**Published:** 2014-02-25

**Authors:** Xu Ji, Yuan Zhu, Mingming Chen, Longxing Su, Anqi Chen, Xuchun Gui, Rong Xiang, Zikang Tang

**Affiliations:** 1 State key Laboratory of Optoelectronic Materials and Technologies, School of Physics and Engineering, Sun Yat-Sen University, Guangzhou 510275, China; 2 Physics Department, Hong Kong University of Science and Technology, Clear Water Bay, Kowloon, Hong Kong

## Abstract

Modulation of grain boundary barrier in ZnO layer by polar liquid, was investigated in ZnMgO/ZnO heterostructures grown by plasma-assisted molecular beam epitaxy. Traditionally, surface adsorbates can only affect the surface atoms or surface electronic states. However, it was found that the electronic conduction property of ZnO far from the surface could be tailored obviously by the polar liquid adsorbed on the ZnMgO surface. Physically, this phenomenon is supposed to be caused by the electrostatical couple between the liquid polarity and the grain boundary barrier in the ZnO layer through crystal polarization field.

ZnO, a wide and direct band gap semiconductor with extraordinary electronic, optical, and chemical properties^1^, has become an attractive material since the realization of ultraviolet laser action at room temperature^2^,^3^. Most of the ZnO films synthesized by ordinary growth techniques are n-type and often have (0001) oriented columnar structures owing to its polar nature^4^,^5^. And the n-type conductivity is significant for transparent conductors and can be influenced by grain boundaries (GBs) around adjacent columnar ZnO crystals through carriers trapping and scattering process. Usually, in polycrystalline ZnO transparency thin-film transistors, the formation of double Schottky barriers in GBs is considered to be the main constraints for device performances. Moreover, the GB potential barrier height in ZnO can be modulated by gate bias^6^,^7^, and can also be suppressed by introducing a ZnMgO layer to form ZnMgO/ZnO heterostructure^8^.

ZnMgO/ZnO polar heterostructures^9^,^10^,^11^,^12^,^13^,^14^, as the pioneer of the discovery of quantum Hall effect in oxides^15^, have attracted intensive research interest due to the novel physical properties that are absent in their individual bulk. It is essential that the polarization fields can dramatically influence the material properties and device behavior^16^ through influencing potential, charge distributions and carrier transport^17^,^18^,^19^. Furthermore, polarization field can be effectively modulated by controlling the layer thickness, defects, composition, other surface coating layers of ferroelectric, such as LiNbO_3_, PbTiO_3_, etc^20^,^21^,^22^. In the LaAlO_3_/SrTiO_3_ heterostructures, for instance, the electron conductivity behaviors at the functional interface have been modulated successfully after the introduction of ploar liquid molecules^23^,^24^,^25^. Also, ZnO/ZnMgO heterostructures have been applied in biosensors by modifying ZnMgO surface with some chemical functional groups^26^,^27^.

This letter concentrates on the modulation of GBs barrier in ZnO layer by the introduction of polar liquid on the surface of ZnMgO layer. Based on room-temperature current-voltage (I-V) test to the ZnMgO/ZnO heterostructure using an in-plane FET device structure, it was found that the carrier transport property in ZnMgO/ZnO heterostructures have been modulated from ohmic to Schottky-like after the introduction of polar liquids. Furthermore, the mechanism of this modulation behavior was discussed in terms of the GBs barrier^7^ that was controlled by coupling effect of the liquid polarization and crystal polarization. It is worth to notice that the double saturation characteristic of I-V curve agrees well with that of the double Schottky diode model. This work offered direct and strong evidence that the GBs barrier and polarization play a key role in the physical property and device performance of ZnO-based polar structures.

## Results

**Figure 1:**
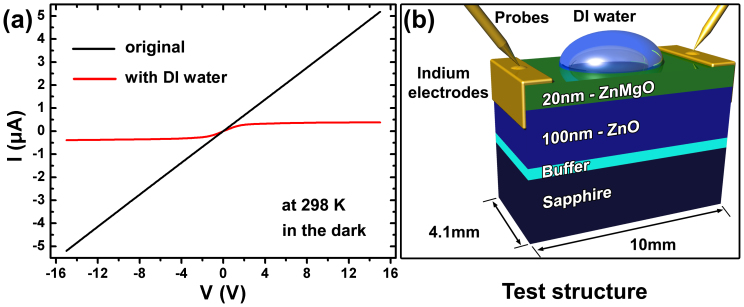
(a) Two-probe *I*–*V* characteristics recorded to the In-contacted ZnMgO/ZnO heterostructure (sample 1) in the dark with 20 μL and without DI water, (b) the schematic diagram of the test structure with DI water.

The transport properties of the ZnMgO/ZnO heterostructure (sample 1) were first investigated by *I-V* characterization. The device structure used in this test is sketched in Figure 1(b), where a piece of rectangle ZnMgO/ZnO sample (10 mm × 4.1 mm) was adopted and indium metal was plated as contact electrodes. Figure 1(a) presents the *I-V* curves of the device before and after placing a droplet of 20 μL DI water at top center of the ZnMgO surface. It can be seen from the *I-V* characteristics that a perfect ohmic behavior was obtained for the sample tested in air, illustrating a metallic conduction of the ZnMgO/ZnO heterostructure. After a water droplet was placed on the ZnMgO surface, the *I-V* curve changed into a typical FET source-drain type current with a strong saturation at high volgtages. Moreover, the *I-V* curve was recovered and exhibited ohmic behavior after all visible water was blown off, which implied the impact of water droplet to the device that worked just as a gate voltage in the device. The electrical resistance of single layer ZnMgO (3.18 × 10^11^ Ohm) is higher than that of heterostructure (about 10^6^ Ohm). Therefore, it can be concluded that the main electrical conduction channel should come from the ZnO layer.

**Figure 2:**
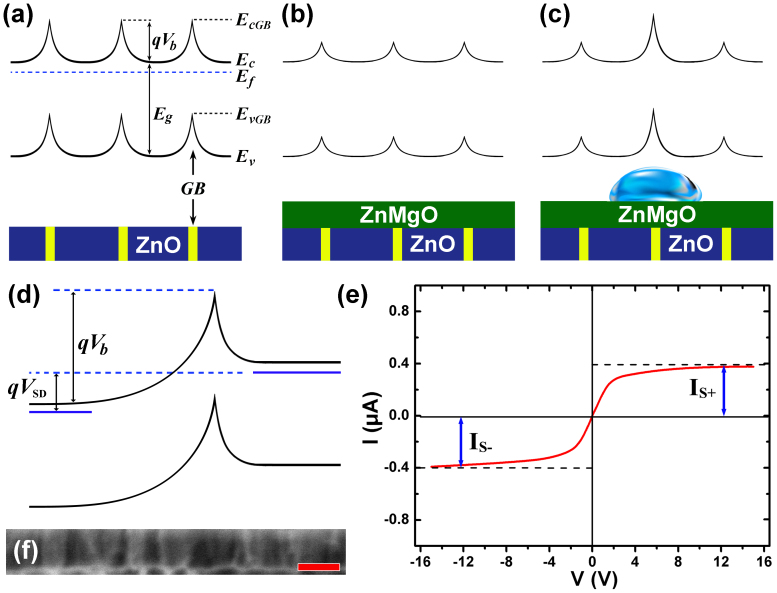
Energy band bending diagram of the ZnO film (a) with GBs, (b) covered by ZnMgO layer, (c) after the introduction of water droplet in the ZnMgO/ZnO system, (d) under applied source-drain voltage *V*_SD_ with water droplet in the ZnMgO/ZnO system; (e) *I-V* curve of the device with 20 μL water on the surface; (f) Cross-section SEM image of the ZnO layer with GBs (scale bar: 100 nm).

Hall measurement was operated to detect the electron behavior of the heterostructure. It gives a sheet carrier density of 1.21 × 10^12^ cm^−2^ and a lower mobility (2.4 cm^2^ V^−1^ s^−1^) in contrast to previous report at room temperature^28^. To account for this result, it was believed that the possible interfacial GBs^7^ barrier scattering and phonon scattering should be responsible for this low mobility^29^. Moreover, the two dimensional electron gas was not formed at the ZnMgO/ZnO heterostructure owe to a poor-quality interface. It is well known that the ZnO film contains lots of columnar crystals that join together by the GBs as shown in the Figure 2(f). Futhermore, large number of defects at the GBs will result in the formation of a wide distribution of deep-level trapping states. These trapping states can trap carriers, which arouse potential energy barriers around the GBs and in turn lead to a band bending around them. As we know, ZnO and its alloy have preferable n-type conductivity arising from the fact that they either have a low valence band maximum or a high conduction band minimum. Naturally, GB traps are dominant by donor-like trap that is positively charged and therefore can capture negative charge. This will generate double Schottky barriers at the condition of electrostatic and thermal equilibrium, as shown in the band diagram of Fig. 2(a). Therefore, the motion of carriers between neighboring columnar crystals will be impeded by the barriers. Whereas, under bias voltage, the carriers transport through the GBs and defect-free columnar crystals are thermionic emission process and drift-diffusion process, respectively^6^. It is reasonable to assume that all the applied voltage drops on the GBs and no voltage drops on single-crystalline ZnO around them, thus the conductivity could be described by following equation^6^: 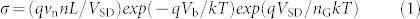
where *n_G_* is the number of GBs in the conduction channel with a length of *L*, *v_n_* is thermal velocity of the electron, *n* is electron density, *V*_SD_ is the applied voltage from source to drain, and *qV*_b_ is the GB energy barrier. The barrier *V*_b_ follows an inversely proportional relationship with *n* as 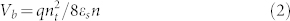
where *n*_t_ and *ε*_s_ are net negative charge in the GB and dielectric constant of ZnO, respectively. As shown in Figure 2(b), when ZnMgO epilayer was grown on lattice-mismatched ZnO layer and was therefore under compressive strain, simultaneously, a piezoelectric field is generated in the ZnMgO layer due to built-in strain. And both the spontaneous and piezoelectric polarization can suppress the GB potential^8^. In addition, according to the equation (2), ZnMgO epilayer could also supply carriers and increase the electron density *n* in the ZnO layer which should suppress the GB barrier more^8^. Furthermore, the I-V test of the heterostructure with DI water was also operated in fluorescent lighting condition. In this case, I-V curve presented weak modulating characteristic, i.e., the electron density *n* in the ZnO increased because of the photo-generated carriers, thus the height of GBs barrier was suppressed.

Hereafter, a water droplet was introduced on the surface of ZnMgO. In molecular perspective, the origin of the water molecule polarization is its distorted tetrahedral of H-O-H with an angle of 104.5°. In the water droplet, the H_2_O molecules are linked together by a very weak hydrogen bonding and the bonding angle is easy to be changed. The polar water molecules have a tendency to align their dipole moments with the effect of electrostatic field and reach a stable state. As a result, the polar field induced by water will weaken the local polarization potential in the ZnO layer and thus reduce the local carrier density in ZnO layer under the water droplet. According to the equation (2), the reduction of carrier density *n* will result in an increase of the GB barrier *qV*_b_. Accordingly, as shown in Figure 2(c), the enhanced double Schottky barriers under the water droplet in the ZnO layer could give a modulation to the *I-V* characterization. Figure 2(d) illustrates the band diagram of a GB under the water droplet with bias voltage. Particularly, an applied source-drain voltage (*V*_SD_) can increase the GB barrier *qV*_b_ on one side. When the *V*_SD_ is small, the low GB barrier makes the conduction channel exhibit a characteristic of resistor; when *V*_SD_ gets large, more electrons will be restricted by increased GB barrier and only limited amount of electrons will be injected through a tunneling effect, as a result, the junction current tends to be saturation. According to the *I-V* result in Figure 1 and/or Figure 2(e), it can be seen clearly that the closest model in spirit to our device is the double Schottky diode model^30^. The total junction current *I* can be expressed as following: 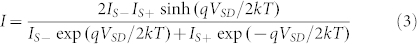
where *I_S−_* and *I_S+_* are the saturation currents as shown in the Figure 2(e). From this expression, the double saturation character of the *I-V* curve can be determined by taking the limits *V_SD_* → ∞ and *V_SD_* → −∞.

**Figure 3:**
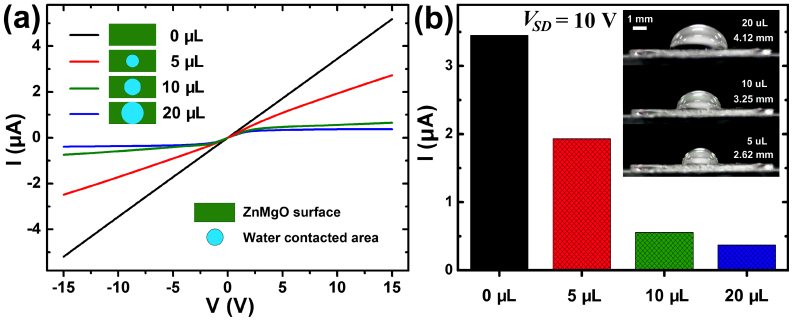
(a) *I-V* curves of sample 1 with different volume of water droplets on the surface of ZnMgO. (b) Corresponding current values under the same bias (10 V) with different volume of water droplets, and the inset show clearly the contacted situation of different water droplets (the values are water droplets' volume and contact diameter, respectively).

The modulation of the DI water on the I-V behavior is obvious. Besides this, there is another significant phenomenon: the currents could be modified conveniently by the contacted area of droplets. As shown in Figure 3(a), for a smaller water droplet (5 μL and 2.62 mm in contact diameter, inset of Figure 3(b)), corresponding smaller contact area (5.39 mm^2^) on the surface of ZnMgO, the current *I* is hard to get saturation along with the increase of *V*_SD_. According to the equation (1), a small number of GBs *n*_G_ under the water caused by a small droplet (5 μL) indicates a high conductivity *σ*, which gives weak conduction modulation effect. While, a bigger droplet, 10 μL, corresponding to larger contacted area (8.30 mm^2^), will surpress conductivity *σ*, as a result, the conduction modulation effect becomes stronger. This relationship can be seen clearly from Figure 3(b) that when the applied bias *V_SD_* was kept at 10 V, the conductive current *I* is inversely proportional to the contacted area of water.

**Figure 4:**
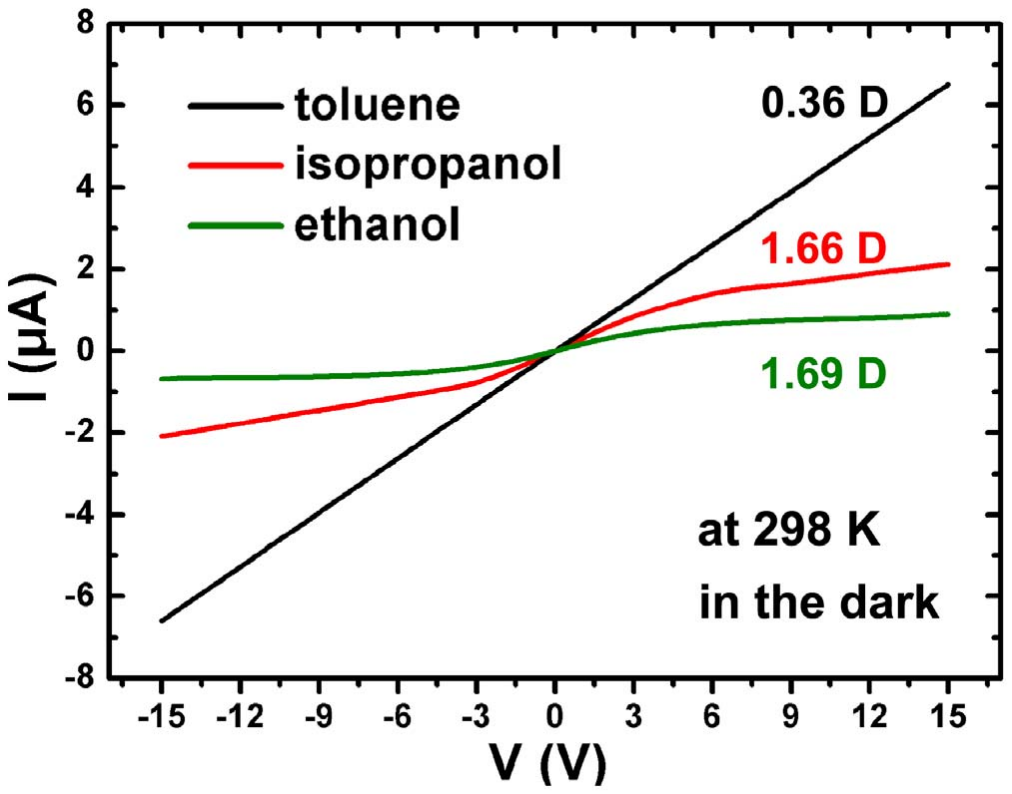
*I–V* characteristics of sample 1 measured with other kinds liquids.

In order to confirm the effect of liquid polarization, a variety of liquids with various polar moments, including ethanol, isopropanol, toluene etc., have been adopted. The dipole moment values of ethanol, isopropanol and toluene are 1.69 D, 1.66 D and 0.36 D, respectively. Because of fast volatile of these organic solvents and their small surface tension on ZnMgO surface, 4.1 mm × 4.1 mm cleanroom wipers (55% cellulose, 45% polyester) were first capped on the center of ZnMgO surface to slow evaporation rate of the organic solvents and fix the contact area. And the size of capping paper is designed to keep the width equal to that of the device. Figure 4 exhibits the *I-V* curves of these tests. All *I-V* curves of the polar liquids, such as ethanol and isopropanol, show the obvious conduction modulation effect. Moreover, the *I-V* curves were recovered to be ohmic behavior after all visible liquids were blown away. However, the weak-polar toluene has no obvious influence to the transport properties of the device. According to the equation (1), for different liquids, except the GB barrier *qV*_b_, all the other parameters are identical. The GB barrier *qV*_b_ that can be modulated by the liquid polarity, i.e., the GB barrier height is proportional to the dipole moment of organic agents, is the key factor to the electrical conduction behavior, as described in Figure 4. In principle, the liquid polarity can suppress the crystal polarization field of ZnO, which in turn enhance the local GB barrier *qV*_b_ through decreasing the mobile carriers density *n* as expressed in the equation (2).

**Figure 5:**
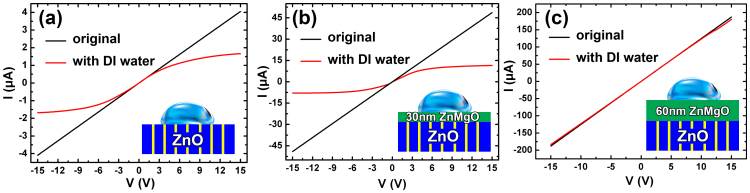
*I–V* characteristics measured to (a) ZnO film (sample 2) and ZnMgO/ZnO heterostructure with ZnMgO thickness (b) 30 nm (sample 3) and 60 nm (sample 4), respectively, in the dark with 20 μL DI water. The insets are schematic diagrams of the main test structures.

To understand the role of ZnMgO coverlayer on the device behavior, the conduction properties of ZnO films and ZnMgO/ZnO heterostructures with different ZnMgO thickness have also been studied. As shown in Figure 5(a), the conduction modulation effect of DI water on ZnO film free of ZnMgO layer is still obvious, which further indicates that the GB's barrier height in the ZnO layer is the key factor for suppressing conduction. Moreover, the resistance of sample 3 (ZnMgO/ZnO with 30 nm ZnMgO coverlayer) calculated from Figure 5(b) is lower than that of sample 2 (ZnO film), i.e., an additional ZnMgO layer can increase the conductivity of the ZnO layer, which further confirmed the statement in Figure 2(b), additional ZnMgO coverlayer can suppress the height of GB barrier in ZnO. Furthermore, a larger ZnMgO thickness will contribute to higher carrier concentration in the ZnO layer, as a consequence, the conductivity of sample 4 (ZnMgO/ZnO with 60 nm ZnMgO coverlayer) (calculated from Figure 5(c)) is higher than that of sample 3. It is worth to mention that the conduction modulation effect of the sample 3 with 30 nm ZnMgO coverlayer is more obvious than that of ZnO film (sample 2). However, the conduction modulation effect in sample 4 almost disappeared, as shown in Figure 5(c), which should owe to the electric screen effect of the thicker ZnMgO to the interaction between liquid polarity and GB barrier in ZnO. Therefore, an optimized ZnMgO thickness is needed for obtaining ZnMgO/ZnO devices that have best sensitivity to polar liquid. Besides, the ZnMgO coverlayer can also prevent direct contaction between ZnO layer and test liquid to protect functional ZnO layer in a practical device.

## Discussion

These results give a solid support that the transport property of ZnMgO/ZnO heterostructures can be successfully controlled through the modulation of GBs barrier in ZnO layer by polar-effect via the surface polar liquid droplet. Moreover, the ZnMgO layer with an optimized thickness can enhance the conduction modulation effect and the conductivity of ZnO film. Furthermore, the device behavior that the GB barrier height is proportional to the dipole moment of organic agents may be exploited to design ZnMgO/ZnO-based sensors for polar liquids.

In conclusion, at the ZnMgO/ZnO heterostructure, it was found that a droplet of polar liquid on the surface of ZnMgO cover-layer can change the local electron transport behavior by influencing the GBs barrier in the ZnO layer. This effect is credited to the change of the crystal polarization induced by the polar nature of the liquids. The polar liquid modulation to crystal polarization, hence the electron density in ZnO layer and further to GBs barrier is the dominant influence factor to the conduction modulation effect. In addition, an optimized coverlayer thickness of ZnMgO is also an important factor to the magnified effect. Significantly, the phenomenon that GBs potential barrier far from surface can be dramatically modulated by polar liquids on the surface sheds a new light on the importance of polarization in understanding the electron transport behavior of polar semiconductors.

## Methods

### ZnMgO/ZnO heterostructures and ZnO film growth

The investigated epitaxial ZnMgO/ZnO heterostructures were grown by plasma-assisted molecular beam epitaxy (PAMBE) on c-plane sapphire substrates in background vacuum of ~1 × 10^−10^ Torr. The growth was monitored by reflection high energy electron diffraction (RHEED) during growth. The sapphire substrate was first annealed at 790°C in the vacuum for 10 min and then in an oxygen plasma for another 10 min. Thin 5 nm MgO and 5 nm ZnO buffer layers were grown on the sapphire substrate in sequence. Based on the thicker MgO buffer layers, 100 nm-thick Zn polarity ZnO functional layer^31^ was deposited at 680°C followed by 15 min in situ annealing at 790°C. Finally, 20 nm-thick Zn_0.7_Mg_0.3_O epitaxial layer was synthesized on the ZnO layer, which was named as sample 1. Moreover, in order to study the role of ZnMgO coverlayer, epitaxial ZnO and ZnMgO/ZnO films with different ZnMgO thickness were also synthesized. ZnO functional layers (1 μm) of these samples were deposited at 550°C followed by 15 min *in situ* annealing at 750°C. ZnO film without coverlayer was named as sample 2. Sample 3 and sample 4 are the ZnMgO/ZnO heterostructures with thickness of Zn_0.7_Mg_0.3_O cover layers of 30 nm and 60 nm, respectively.

### Measurement of electrical properties of the samples

Using indium as contact electrodes, two-probe *I-V* characteristics of ZnMgO/ZnO heterostructure were measured at room temperature in the dark using Agilent Technologies 4155C semiconductor parameter analyzer, and Hall effect was tested in the Van der Pauw configuration by Lake Shore 7700A Hall effect measurement system to detect the electric properties of the samples.

### Characterization of the cross-section information of the ZnO layer of sample 1

Sample 1 was characterized by field emission scanning electron microscopy (FE-SEM; S-4800) to obtain the cross-section information.

## Author Contributions

Z.T. and Y.Z. directed the project. X.J. proposed and designed the project, M.C. and L.S. carried out the experiment of film growth, A.C. carried out the Hall test, X.G. analyzed the data through discussions with R.X. All the authors discussed the results and contributed to the final version of the manuscript.
